# Concentration Scaling on Linear Viscoelastic Properties of Cellular Suspensions and Effects of Equilibrium Phase Behavior

**DOI:** 10.3390/ijms24044107

**Published:** 2023-02-18

**Authors:** Geng-Xin Xu, Xue-Feng Yuan, Qing-Song Liu, Howard Wang

**Affiliations:** 1 Institute for Systems Rheology, Guangzhou University, No. 230 West Outer Ring Road, Higher Education Mega-Center, Panyu District, Guangzhou 510006, China; 2 Neutron Science Platform, Songshan Lake Materials Laboratory, Dongguan 523808, China

**Keywords:** cellular suspensions, nanocrystals, liquid crystalline polymer, rheometric characterisation, time-concentration superposition principle

## Abstract

Concentration scaling on linear viscoelastic properties of cellular suspensions has been studied by rheometric characterisation of *Phormidium* suspensions and human blood in a wide range of volume fraction under small amplitude oscillatory shear experiments. The rheometric characterisation results are analysed by the time-concentration superposition (TCS) principle and show a power law scaling of characteristic relaxation time, plateau modulus and the zero-shear viscosity over the concentration ranges studied. The results show that the concentration effect of *Phormidium* suspensions on their elasticity is much stronger than that of human blood due to its strong cellular interactions and a high aspect ratio. For human blood, no obvious phase transition could be observed over the range of hematocrits studied here and with respect to a high-frequency dynamic regime, only one concentration scaling exponent could be identified. For *Phormidium* suspensions with respect to a low-frequency dynamic regime, three concentration scaling exponents in the volume fraction Region I ($0.36\leq \phi /\phi_{ref}\leq 0.46$), Region II ($0.59\leq \phi /\phi_{ref}\leq 2.89$) and Region III ($3.11\leq \phi /\phi_{ref}\leq 3.44$) are identified. The image observation shows that the network formation of *Phormidium* suspensions occurs as the volume fraction is increased from Region I to Region II; the sol-gel transition takes place from Region II to Region III. In combination with analysis of other nanoscale suspensions and liquid crystalline polymer solutions reported in the literature, it is revealed that such a power law concentration scaling exponent depends on colloidal or molecular interactions mediated with solvent and is sensitive to the equilibrium phase behaviour of complex fluids. The TCS principle is an unambiguous tool to give a quantitative estimation.

## 1. Introduction

Human blood and cyanobacteria in the natural environment are cellular suspensions composed of biofunctional cells and nutrient solution [1]. Blood flows through arteries, capillaries and veins to maintain the body’s metabolism and other physiological functions [2,3]. As the most primitive autotrophic organisms on earth, cyanobacteria play a key role in the oxidative environment of the earth, and recently shown its great potential in the development of renewable energy and natural anti-cancer drugs [4,5]. On the other hand, the savage growth of cyanobacteria could result in environmental pollution that seriously endangers the natural environment, organisms and human health [6]. From the management of cyanobacterial blooms, the development of green energy based on cyanobacterial biomass, and the processing of natural medicines, to the regulation of cardiovascular diseases, all requirements for a better understanding of the rheological properties of cellular suspensions from a unified principle of multiple-scale complex fluid dynamics.

Human blood is mainly composed of biconcave disc-shaped red blood cells (RBCs) with a diameter of about 8 μm and a thickness of 2 μm, a small number of white blood cells and plasma containing proteins. Driven by a net attractive force, RBCs could self-assemble into rouleaux, that in turn can be disrupted under sufficiently strong shear or extensional flow. For most studies, plasma is usually considered a Newtonian fluid. The viscoelastic properties of blood are mainly caused by the rouleaux formation and deformable RBCs themself [2,3,7]. Thurston [2] characterized the viscoelastic properties of blood. The effects of anticoagulant, strain rate and hematocrit on hemorheology are also studied but mainly under steady shear flows [2,8,9,10]. The experimental results of Sousa et al. [10] show that anticoagulants have no effect on the rheological properties of blood. Cokelet and Meiselman [11] characterized the shear viscosity of blood with a hematocrit of 20–50% and found that the change of viscosity with hematocrit is more pronounced at a low shear rate (0.11 $s^{-1}$) than that at a high shear rate (20 $s^{-1}$), above which the blood behaves like Newtonian fluid. Tomaiuoloe et al. [12] characterized the linear viscoelastic properties of blood with three hematocrits (45%, 75%, 90%) in a small frequency range (0.1∼30 rad/s). Under a uniaxial extensional experiment, Sousa et al. [13] measured the extensional relaxation time of the blood samples extracted from thirteen healthy people with a hematocrit range of 38.7∼46.3%. No clear concentration scaling could be identified due to significant errors in their measurements.

As a kind of filamentous cyanobacteria, *Phormidium* suspension has recently attracted much attention owing to its lower toxicity and potential for various industrial applications [14,15,16]. *Phormidium* cells are connected by septal junction along their filamentous contour direction and wrapped together by the cell wall providing considerable mechanical strength [17]. The theoretical framework for studying such a filamentous suspension can be traced back to the rigid rod-like molecular theory proposed by Onsager [18]. It predicts a transition from an equilibrium isotropic phase to an anisotropic liquid crystal phase as the volume fraction of rod-like molecules with a certain aspect ratio is increased. Onsager’s theory is also applicable in studies of phase transition from nanocrystalline cellulose (CNC [19], cellulose/AmimCl [20] and hydroxypropylcellulose (HPC) [21]) to micron scale cellulose and bio-particle (Cellulose Whiskers [22], fd virus [23] and Sacran solutions [24]). Phase transitions of structural fluids could affect their rheological properties. For example, the zero-shear viscosity $\eta_{0}$ or modulus ($G^{\prime }$ and $G^{\prime \prime }$) approaches a maximum value when a phase transition occurs [19,25]. However, the maximum value of those rheometric properties could be obscured by the polydispersity of colloidal dispersions.

Overall, cellular interactions determine equilibrium phase behaviour and rheological characteristics of cellular suspensions. Equilibrium and nonequilibrium properties of blood are similar to particulate solutions with a weak net attraction. A strong interaction between cells can form *Phormidium* like solutions, which may behave like a rod or semi-flexible filamentous suspensions. Thus, a comprehensive comparison of the rheological characteristics among micron-scale cellular suspensions, nanoscale suspensions and liquid crystalline polymer solutions will gain a better understanding of the dependence of rheological properties on cellular or molecular interactions in order to regulate the rheological properties by formulation design of complex fluids. In this study, the linear viscoelastic properties of human blood and *Phormidium* suspensions over a wide range of concentrations are reported. Since no unified theory is available yet to predict the concentration scaling of cellular suspensions, their linear viscoelastic properties are analyzed by the time-concentration superposition (TCS) principle. Through comparison with the results from TCS analysis of the linear viscoelastic data available in the literature for other colloidal dispersions and polymer solutions, it will be demonstrated that TCS is a powerful method to quantitatively estimate the concentration scaling exponents on linear viscoelastic properties of complex fluids. The experimental results are reported in Section 2. The data analysis and discussion are presented in Section 3. The materials and experimental methodology are illustrated in Section 4. The conclusion is drawn in Section 5.

## 2. Results

### 2.1. Microstructure Characterization

**Figure 1:**
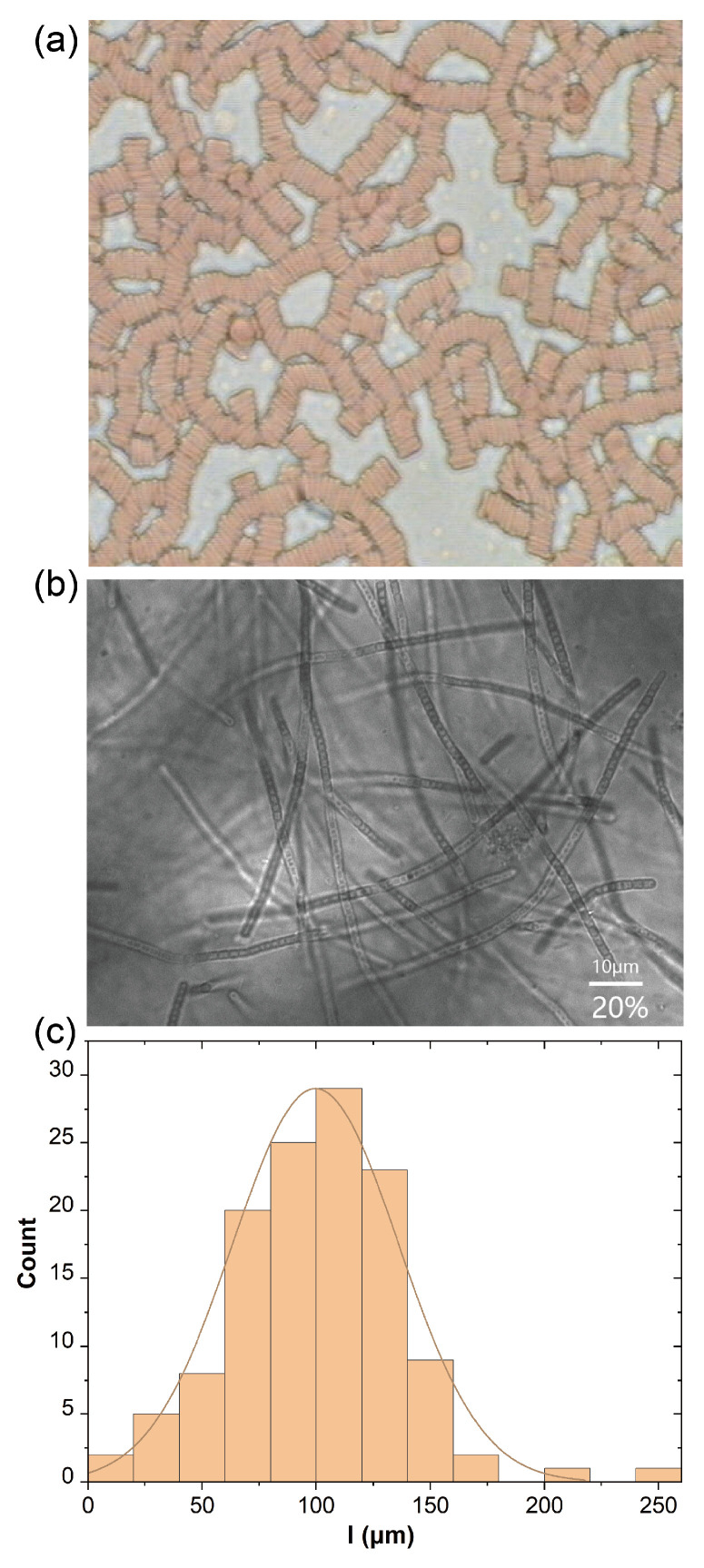
(**a**) A typical self-assembled RBC structure of human blood (the diameter of RBC (8 μm) as a scale); (**b**) a typical structure of *Phormidium* suspensions; (**c**) the length distribution of *Phormidium* suspensions.

Figure 1a,b show a typical mesoscopic structure of human blood and *Phormidium* suspension. As the RBC concentration increases, RBCs would self-assemble into rouleaux and form a weak gel-like network structure. Similarly, with an increase in concentration, *Phormidium* would overlap and form a network structure in its solution. Upon further increases of concentration, an anisotropic phase emerges and will dramatically affect its concentration scaling of rheological properties as discussed later. From image analysis, it has been found that *Phormidium* has approximately a constant diameter of d=2 μm and variable lengths. Its length distribution is shown in Figure 1c. In a similar way to the characterization of linear polymer molecular mass distribution, its number averaged length $L_{n}$ could be calculated as $L_{n}=\frac{\sum_{i}^{}N_{i}l_{i}}{\sum_{i}^{}N_{i}}=100.4$ μm and the second order averaged length is $L_{w}=\frac{\sum_{i}^{}N_{i}l_{i}^{2}}{\sum_{i}^{}N_{i}l_{i}}=114.1$ μm, where $l_{i}$ is the average length of the $i^{th}$ portion *Phormidium* and $N_{i}$ is a number of that fraction. Thus, the polydispersity index is $d=L_{w}/L_{n}=1.14$, which could be approximately considered as a narrowly distributed *Phormidium* suspension.

### 2.2. Linear Viscoelasticity

**Figure 2:**
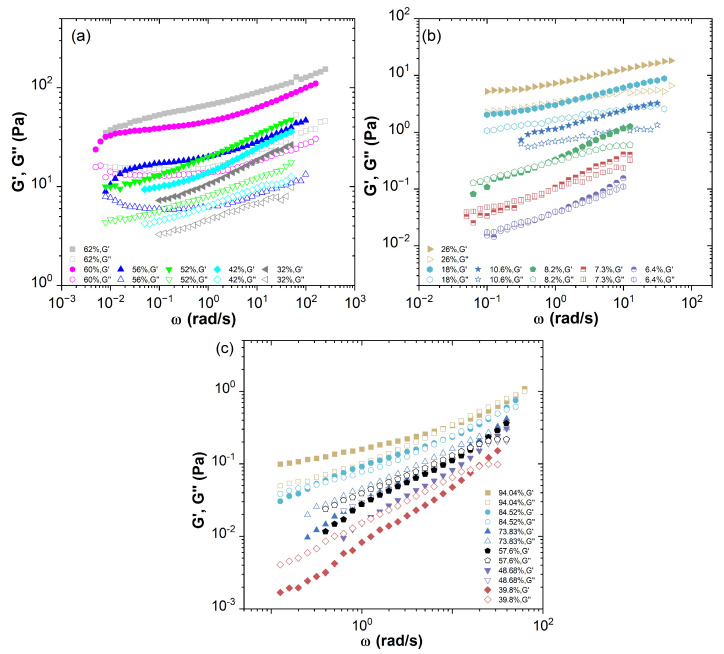
Plots of the storage modulus $G^{\prime }$ and the loss modulus $G^{\prime \prime }$ against frequency obtained from small amplitude oscillatory shear experiment in the linear viscoelastic region. (**a**,**b**) for the *Phormidium* suspensions in various volume fractions and (**c**) for the human blood samples in various volume fractions.

*Phormidium* suspensions and human blood with various volume fractions were characterised under an oscillatory shear flow at a constant small strain amplitude in the linear viscoelastic region and over a range of frequencies. The results are shown in Figure 2a–c. With the increase in volume fraction, the storage modulus $G^{\prime }$ and loss modulus $G^{\prime \prime }$ of the cellular solutions are increased and the slopes of the frequency sweep curves are decreased. The results show a strong concentration dependence. For a dilute *Phormidium* suspension (6.4%≤ϕ≤10.6%), the intersection of the storage modulus $G^{\prime }$ and the loss modulus $G^{\prime \prime }$ slightly shifts to the low-frequency region. At a higher volume fraction in a range of 10.6%<ϕ≤52%, there is no intersection over the frequency range measured experimentally. At an even higher volume fraction range (52%<ϕ≤56%), a near plateau of $G^{\prime }$ appears at a lower frequency region along with a minimum of $G^{\prime \prime }$.

For the human blood samples only with much higher hematocrit ($\phi_{h}\geq 84.52\%$), the storage modulus $G^{\prime }$ becomes comparable or larger than the loss modulus $G^{\prime \prime }$ over the entire frequency range measured. Otherwise, the loss modulus $G^{\prime \prime }$ dominates over the storage modulus $G^{\prime }$. With the decrease in hematocrit, the elastic effects become even weaker, this is consistent with the results of Tomaiuolo et al. [12]. Obviously, the concentration effect of *Phormidium* suspensions on their elasticity is much stronger than that of blood samples due to its strong cellular interactions and a high aspect ratio. The difference in their concentration scaling will be analysed by the TCS procedure as shown below.

## 3. Discussion

### 3.1. Time-Concentration Superposition Analysis

**Figure 3:**
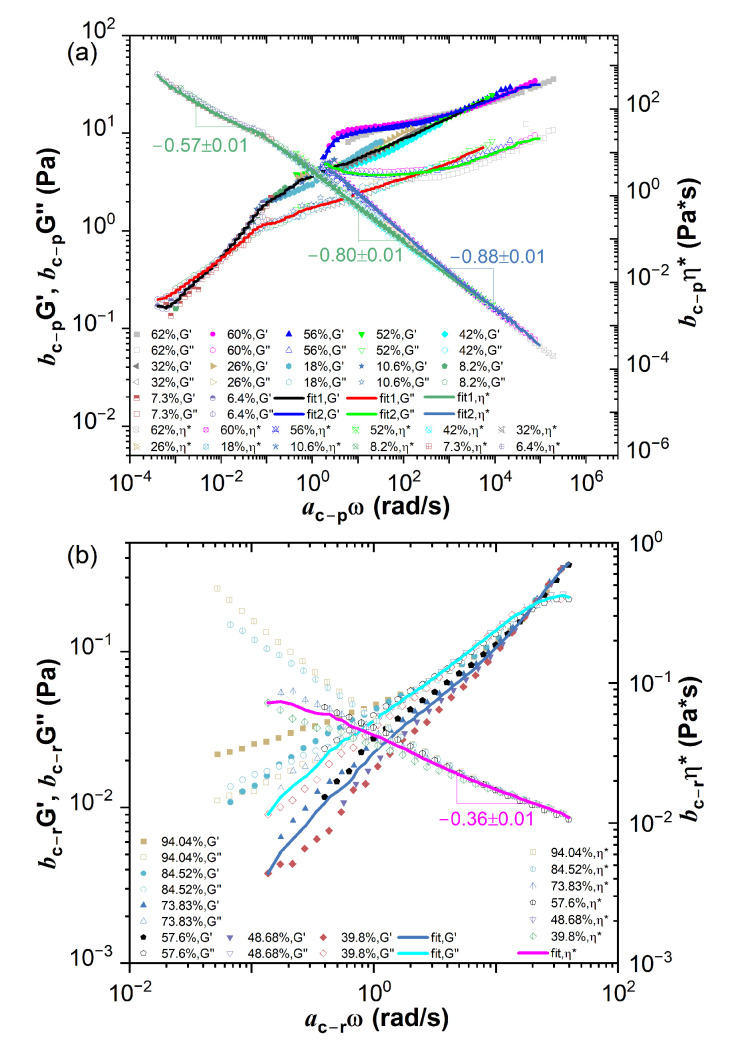
(**a**) The master curve of *Phormidium* suspension with $\phi_{ref}=18\%$, (**b**) the master curve of blood with $\phi_{href}=57.6\%$. The subscript of the shifting factors p represents *Phormidium* suspension, r represents human blood with RBC. The fitted master curves are shown in the lines.

Similar to the time-temperature superposition (TTS) principle, the concentration dependence of the linear viscoelastic properties of cellular suspensions could be analyzed by applying the TCS procedure to the data presented in Figure 2. As studying concentration scaling of polymer solutions [26], an arbitrary volume fraction is selected as a reference concentration as $\phi_{ref}=18\%$ for *Phormidium* suspension and $\phi_{href}=57.6\%$ for human blood. The master curves of the storage modulus $G^{\prime }\left(\omega \right)$ and the loss modulus $G^{\prime \prime }\left(\omega \right)$ could be constructed by shifting them horizontally and vertically by a factor $a_{c}$ and $b_{c}$, respectively. The result is shown in Figure 3a,b along with the shifted complex viscosity $\eta^{*}\left(\omega \right)$. Since the rheometric properties of cellular suspensions involve typically multiple and well-separated dynamic lengths and time scales, the TCS could only be realized either in the low-frequency dynamic regime or in the high-frequency dynamic regime. The complex viscosity curves of the human blood samples could only be superimposed into a master curve in the high-frequency dynamic regime with a scaling exponent −0.36±0.01. For the master curves of *Phormidium* suspensions, three scaling exponents corresponding to the low, middle and high-frequency region, respectively, −0.57±0.01, −0.80±0.01 and −0.88±0.01, could be identified. Therefore due to the dynamic time scale separation, the better superimposed linear viscoelastic data of human blood over the high-frequency dynamic regime would inevitably result in the less well superimposed in the low-frequency dynamic data, and vice versa for *Phormidium* suspensions.

**Figure 4:**
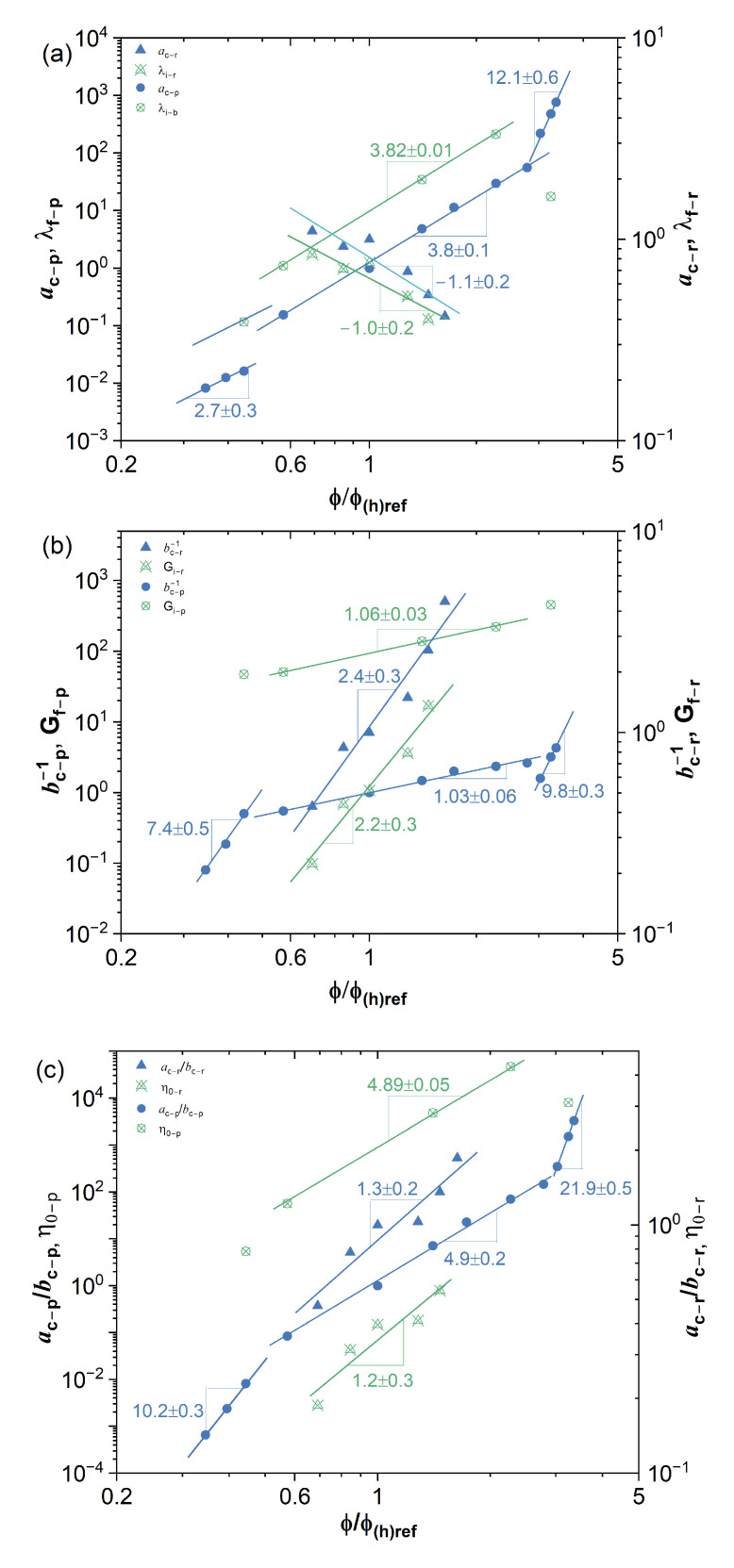
The concentration scaling of the estimated properties (hollow symbols, $\lambda_{f}$, $G_{f}$, $\eta_{0}$) and the shifting factors (solid symbols, $a_{c}$, $b_{c}^{-1}$, $a_{c}/b_{c}$), (**a**) is for the mean characteristic time, (**b**) is for the mean modulus, (**c**) is for the zero-shear viscosity. $\phi_{ref}=18\%$ for *Phormidium* suspension (circle), $\phi_{href}=57.6\%$ for human blood (triangle).

The master curves could be fitted by the multi-mode Maxwell model $G^{\prime }\left(\omega \right)=\sum_{i}^{N}\frac{G_{i}\lambda_{i}^{2}\omega^{2}}{1+\lambda_{i}^{2}\omega^{2}}$ and $G^{\prime \prime }\left(\omega \right)=\sum_{i}^{N}\frac{G_{i}\lambda_{i}\omega }{1+\lambda_{i}^{2}\omega^{2}}$ using IRIS Rheo-hub software [27], where $G_{i}$ and $\lambda_{i}$ are the modulus and relaxation time of the $i^{th}$ mode of the Maxwell model; ω is the angular frequency; *N* is a total number of the modes and is optimized by IRIS software in the fitting Maxwell model, typically *N* = 4 for the human blood samples and *N* = 8∼11 for the *Phormidium* suspension samples. Table 1 lists some typical fitted results. The zero-shear viscosity $\eta_{0}$ of the cellular suspensions at the previously specified reference concentration could be calculated by the fitted data as $\eta_{0}=\sum_{i}^{N}G_{i}\lambda_{i}\omega +\eta_{s}$, where $\eta_{s}$ is the viscosity of solvent. A mean characteristic time $\lambda_{f}$ could be defined by taking an average of the multi-mode relaxation spectrum as $\lambda_{f}=\frac{\sum_{i}^{N}G_{i}\lambda_{i}}{\sum_{i}^{N}G_{i}}$, and a mean modulus is then estimated as $G_{f}=\frac{\eta_{0}}{\lambda_{f}}$. A similar analysis is then applied with respect to all other reference concentrations in order to evaluate the concentration scaling of these estimated properties. The results are shown in Table 2, from which the power law concentration scaling of the mean characteristic time, the mean modulus and the zero-shear viscosity could be obtained as shown in Figure 4a–c, respectively. The scaling exponents are in good agreement with the results estimated by TCS.

The previous analysis of polymer solutions [26] shows that the concentration scaling of the shifting factors $a_{c}$ and 1/$b_{c}$ is equivalent to the concentration scaling of the mean characteristic time $\lambda_{f}$ and the mean characteristic modulus $G_{f}$, respectively. The ratio of these two shifting factors $a_{c}/b_{c}$ is related to viscosity. As shown in Figure 4, the dependence of the shifting factors on concentration exhibits the same power law scaling as the estimated properties over a certain range of the volume fraction as, $a_{c}\left(\phi ;\phi_{ref}\right)=a_{c}\left(\phi \right)/a_{c}\left(\phi_{ref}\right)={\left(\frac{\phi }{\phi_{ref}}\right)}^{\alpha }$, $b_{c}\left(\phi ;\phi_{ref}\right)=b_{c}\left(\phi \right)/b_{c}\left(\phi_{ref}\right)={\left(\frac{\phi }{\phi_{ref}}\right)}^{\beta }$ and $a_{c}/b_{c}={\left(\frac{\phi }{\phi_{ref}}\right)}^{\alpha -\beta }$, where α, β and α−β are the concentration scaling exponent of characteristic time, modulus and the zero-shear viscosity, respectively.

### 3.2. Effects of Equilibrium Phase Behavior on Concentration Scaling

The effects of equilibrium phase behaviour on the concentration scaling of the rheological properties of cellular solutions are evident as shown in Figure 4. For the human blood samples with respect to its high-frequency dynamic regime, no obvious phase transition could be observed over the range of hematocrits studied here and only one scaling exponent could be identified. However, for *Phormidium* suspensions, there are three concentration scaling exponents in the volume fraction Region I ($0.36\leq \phi /\phi_{ref}\leq 0.46$), Region II ($0.59\leq \phi /\phi_{ref}\leq 2.89$) and Region III ($3.11\leq \phi /\phi_{ref}\leq 3.44$), respectively. The image observation reveals that from Region I to Region II, the network formation of liquid crystalline domains occurs in *Phormidium* suspensions as the volume fraction is increased. The sol-gel transition takes place from Region II to Region III. Table 3 lists the concentration scaling exponents estimated by the TCS procedure for a wide range of complex fluids, including *Phormidium* suspension in different phases, human blood sample, double-stranded DNA (dsDNA) stabilized single-wall carbon nanotube (SWCNT) dispersion [28], hydroxypropylcellulose (HPC) in ionic liquid (IL) solution [21], aqueous sulfonated cellulose nanocrystal (CNC) suspension [19], monodisperse polybutadiene (PB) in phenyloctane (PHO) binary solution (PB-PHO), highly polydisperse and high molecular weight polyacrylamide (18 M PAAm) in aqueous binary solution [26].

The results show that similar to polymer solutions (e.g., PB-PHO, 18M PAAm), the rheometric properties of colloidal suspensions also exhibit a power law concentration scaling. The scaling exponent is affected by the fundamental hierarchical molecular or particulate forces mediated by solvent, including excluded volume (particulate shape, size and size distribution), chain flexibility, van der Waals, electrostatic, hydrogen-bonding, hydrophobic and other interactions. There is considerable ambiguity in theoretically predicting the concentration scaling of linear viscoelastic properties of entangled flexible polymer solutions. For example, de Gennes’ single parameter scaling theory [29] predicts the power law concentration scaling exponent of the zero-shear viscosity for θ solvent polymer solution as 6.8 and for good solvent polymer solution as 4.5. Whereas under the two-parameter scaling approximation [30] to account for the possible different concentration dependence of the tube diameter and the correlation length, it predicts the same scaling exponent for θ solvent polymer solution as 5.2 and for good solvent polymer solution as 4.5. Obviously, the outcomes are sensitive to the actual solvent quality, which relates to how the mean radius of gyration of the molecular chain is scaled with the molecular weight and has to be pre-determined by other experimental or computational methods. For semi-dilute and concentrated (isotropic) rodlike polymer solutions, Doi and Edwards [31,32] approximated the excluded-volume interactions of rods by assuming that each rod is confined by its neighbouring rods to a tube-like region, is analogous to the tube model for modelling flexible entangled polymer solutions. Their molecular theory predicted that the characteristic rotational time of the confined rod $\lambda_{D}$ and the zero-shear viscosity $\eta_{0}$ are scaled with concentration (ϕ) and molecular weight (*M*) as $\lambda_{D}∼\phi^{2}M^{7}$ and $\eta_{0}∼\phi^{3}M^{6}$, respectively. The prediction is in reasonably good agreement with the experimental results of the *ideal* rodlike polymer solutions with relatively low concentration, low molecular weight and low aspect ratio. However for the *non-ideal* stiff-chain polymer solutions in relatively high concentration, the experimental results show that the power law scaling exponent of the zero-shear viscosity could reach as high as 8 with concentration and with molecular weight, respectively, possibly even higher for wormlike polymer solutions with high concentration [33,34]. The deviations are mainly due to the oversimplifications in the deviation of the molecular theory. Obviously, the present molecular theories cannot quantitatively account for many *non-ideal* factors yet, including finite molecular weight, chain flexibility, polydispersity, hierarchical molecular or particulate forces, possible phase transitions in different temperature and concentration, the effect of *defects* in orientational order etc. TCS provides an alternative and direct method to implicitly account for all the above effects and could estimate the scaling exponent in an unambiguous way.

A weak attraction between RBCs in human blood results in the rouleaux formation and flocculation of RBCs. Thus, there is significant concentration dependence of $G^{\prime }\left(\omega \right)$ and $G^{\prime \prime }\left(\omega \right)$ of human blood in low-frequency regions. A suitable TCS is to shift the data of $G^{\prime }\left(\omega \right)$ and $G^{\prime \prime }\left(\omega \right)$ with respect to a high-frequency dynamic regime. As a consequence, the concentration scaling exponent of the mean characteristic time (α) is negative, a general feature of such a shifting choice, and is consistent with the scaling exponent estimated from the fitting of the multi-mode Maxwell model as shown in Figure 4. It indicates that the mean characteristic time of human blood in a high-frequency dynamic regime is decreased with increasing cellular concentration, likely due to the long-range cellular interactions being screened. Whereas for all the other suspension solutions analyzed here, their rheometric data was superimposed with respect to a low-frequency dynamic regime, the concentration scaling exponents of their mean characteristic times are all positive. Other concentration scaling exponents of human blood, −β, α−β, are positive and are in line with those of all the other suspension solutions.

The effects of equilibrium phase behaviour on the concentration scaling of rheometric properties are also evident in other colloidal suspensions. According to a study of Ao et al. [28], the rheologically observed percolation occurs in Region II semi-dilute dsDNA-SWCNT nanoscale rod dispersions with an aspect ratio of about 600, probably due to formation of a network of liquid crystalline domains. With further increase in volume fraction, dsDNA-SWCNT rod dispersions undergo a sol-gel transition into a gel phase in Region III, over which the concentration scaling exponents of characteristic time (α), characteristic modulus (−β) and the zero-shear viscosity (α−β) are nearly double to those in Region II. The trend is similar to what was observed in *Phormidium* suspensions. In Region II, the concentration of CNC nanocrystal suspensions with an aspect ratio of about 10–20 is in a biphasic region with the coexistence of isotropic and liquid crystal domains [19]. In such a biphasic region, its concentration scaling exponents of characteristic time (α) and the zero-shear viscosity (α−β) are higher than those of *Phormidium* suspension and dsDNA-SWCNT rod dispersion. Whereas its scaling exponent of characteristic modulus (−β) is smaller than those of the other two suspension solutions. The concentration region of HPC lyotropic liquid crystalline polymer solution shown in Table 3 is below its concentration of sol-gel transition and liquid crystal transition [21]. Its concentration scaling exponent is smaller than those of the monodisperse PB-PHO polymer solution, close to those of the polydisperse 18M PAAm polymer solution. It is probably due to its liquid crystalline polymer nature and polydispersity, which could blur the biphasic region of nanocrystal suspensions.

## 4. Materials and Methods

### 4.1. Materials

The Cyanobacterium *Phormidium* sp. FACHB 238 was obtained from a Freshwater Algae Culture of Hydrobiology Collection at the Institute of Hydrobiology, Chinese Academy of Sciences (Wuhan, China) and made available from Songshan Lake Materials Laboratory. The formulation of its culture medium is 1500 mg/L ${\mathrm{NaNO}}_{3}$, 40 mg/L $K_{2}{\mathrm{HPO}}_{4}$, 75 mg/L ${\mathrm{MgSO}}_{4}$ · $7H_{2}O$, 36 mg/L ${\mathrm{CaCl}}_{2}$ · $2H_{2}O$, 6 mg/L citric acid ($C_{6}H_{8}O_{7}$), 6 mg/L ammonium ferric citrate $\left(C_{6}H_{8}{\mathrm{FeNO}}_{7}\right)$, 1 mg/L EDTA · ${\mathrm{Na}}_{2}$, 20 mg/L ${\mathrm{Na}}_{2}{\mathrm{CO}}_{3}$, 1 ml/L A5 trace metal solution. The A5 trace metal solution contaions 2860 mg/L $H_{3}{\mathrm{BO}}_{3}$, 1860 mg/L ${\mathrm{MnCl}}_{2}$ · $4H_{2}O$, 220 mg/L ${\mathrm{ZnSO}}_{4}$ · $7H_{2}O$, 390 mg/L ${\mathrm{Na}}_{2}{\mathrm{MoO}}_{4}$ · $2H_{2}O$, 80 mg/L ${\mathrm{CuSO}}_{4}$ · $5H_{2}O$, 50 mg/L $\mathrm{Co}{\left({\mathrm{NO}}_{3}\right)}_{2}$ · $6H_{2}O$. A dilute *Phormidium* suspension sample was concentrated by a centrifuging method. Firstly the sample was centrifuged at 4339 rpm for 5 min. Its supernatant was then taken away and an appropriate amount of fresh culture solution was added to the precipitation. The same procedure was repeated three times. Finally, a quantitative volume of the culture medium was added into the precipitation to make a required volume fraction ϕ of *Phormidium* solution from dilute (ϕ=6.4%) to concentrated (ϕ=62%) suspensions.

A blood sample was drawn from a healthy volunteer and collected into a 10 mL vacutainer with 0.12 mL K3 EDTA. The hematocrit $\phi_{h}$ of this blood is 48.60%. Similar to the treatment of *Phormidium* suspension, RBCs of the human blood samples were firstly isolated from its autologous plasma solution by centrifugation (25,000 rpm for 10 min) and then re-suspended back to the autologous plasma solution to make a series of samples with hematocrit $\phi_{h}$ from 39.8% to 94.04%. All tests were carried out within 24 H from venipuncture.

### 4.2. Rheometry

The rheological properties of *Phormidium* suspension were measured at a constant temperature of $25{\;}^{\circ }$C using an ARES rheometer (TA Instruments, New Castle, DE, USA). *Phormidium* suspensions of low concentration (ϕ≤10%) were measured by using concentric cylinder cell (diameter = 27.7 mm and the gap of the concentric cylinders is 5.847 mm). A cone-plate fixture (diameter = 50 mm, cone angle radian = 0.04 rad) was used for the samples with higher concentrations. The experimental spacing of the cone-plate fixture was set to 0.0492 mm. The lower plate is an advanced Peltier system (APS) and connected to a ThermoCube Model 10-300 (115/230 V, 50/60 Hz) water bath system, which can accurately control the experimental temperature at ± 0.2 ${}^{{}^{\circ}}$C. All blood samples were measured by using a double wall Couette cell: 27.95 mm (inside cup diameter), 29.5 mm (inside bob diameter), 32 mm (outside bob diameter), 34 mm (outside cup diameter), and a bob length of 38.05 mm at the body temperature of $37{\;}^{\circ }$C. The cellular suspension samples were pre-sheared with a large amplitude (>500%) oscillatory (1 Hz) shear flow for at least 5 min in order to maintain a consistent initial equilibrium state for characterization of their linear viscoelastic properties.

The linear viscoelastic properties of cellular suspensions were characterized under oscillatory shear flow. Firstly, the storage modulus $G^{\prime }$ and loss modulus $G^{\prime \prime }$ of the testing sample were measured by scanning a range of strain amplitude from 1000% to 0.05 % under a constant frequency 1 Hz oscillatory shear flow. The linear viscoelastic region of testing samples was identified by finding a range of the strain amplitude over which there was little chance of the storage modulus $G^{\prime }$ and loss modulus $G^{\prime \prime }$. The frequency dependence of $G^{\prime }$ and $G^{\prime \prime }$ of *Phormidium* suspensions and human blood with various volume fractions were then characterized by scanning a range of frequencies from 100 Hz to 0.01 Hz under a constant small strain amplitude (typically less than 2%) oscillatory shear flow in the linear viscoelastic region. The measurements were repeated at least twice to check the consistency of the experimental data. Further details of rheometric characterization techniques could be found in textbooks, e.g., [1].

### 4.3. Optical Microscopy

A Nikon microscope with 50–60 times magnification was used in the transmission mode to image the equilibrium mesoscopic structure of human blood and *Phormidium* suspensions filled in a coverslip of 8 mm × 22 mm × 0.5 mm. The *Phormidium* size distribution was obtained by measuring individual particle lengths in its dilute solution through image analysis.

## 5. Conclusions

In the present study, the concentration scaling on linear viscoelastic properties of cellular suspensions has been studied by rheometric characterisation of *Phormidium* suspensions and human blood under small amplitude oscillatory shear experiments. The results show a concentration scaling of characteristic relaxation time, plateau modulus and zero-shear viscosity across a range of concentrations. The concentration scaling of weakly flocculated human blood is only applicable with respect to a high-frequency dynamic regime. As a consequence, the power law exponent of its characteristic relaxation time is negative. It reflects the fact that the characteristic relaxation time over the high-frequency dynamics is decreased with an increase in RBC concentration, likely due to the screening effect of cellular interactions. The scaling exponents of its plateau modulus and zero-shear viscosity remain positive. The concentration scaling of micron-scale filamentous *Phormidium* suspensions is similar to those of nanoscale suspensions and liquid crystalline polymers. The actual power-law scaling exponents of their rheometric properties depend on colloidal or molecular interactions mediated with solvent, hence in turn very sensitive to their phase behaviour including isotropic phase, liquid crystal phase, transitional biphasic phase, gel phase etc. Although no unified theory yet could quantitatively account for the effects of colloidal size and aspect ratio, polydispersity, the flexibility of filamentous suspensions, various colloidal or molecular interactions, equilibrium phase behaviour etc., and to accurately predict such a power law concentration scaling of complex fluids, the present work demonstrates that TCS not only could identify whether a power law scaling may exist or not but also unambiguously estimate the concentration scaling exponents of linear viscoelastic properties for a range of colloidal dispersions as well as liquid crystalline polymer solutions. It will provide a general framework to systematically analyze all the effects on the concentration scaling of complex fluids as long as the data of molecular or colloidal characterization and rheometric characterization are available. The outcomes would facilitate the development of a unified scaling theory. In a different direction of the following up work, the TCS method could be extended in studying concentration scaling of nonlinear rheological properties of complex fluids, which is of much significance in engineering prediction and optimization of complex fluids through molecular and formulation design.

## Figures and Tables

**Table 1:** The typical relaxation spectrum of the master curve of *Phormidium* suspension with $\phi_{ref}=18.0\%$ and human blood with $\phi_{href}=57.60\%$ fitted by using IRIS Rheo-hub software [27]. The mean characteristic time $\lambda_{f}$, the mean modulus $G_{f}$ and the zero-shear viscosity $\eta_{0}$ are also estimated and listed.

Mode	$G_{i}$ (Pa)	$\lambda_{i}$ (s)	$\eta_{i}=G_{i}\lambda_{i}$ (Pa·s)
	*Phormidium* suspension with $\phi_{ref}=18.0\%$		
1 2 3 4 5 6 7 8 9 10 11 Solvent Estimated	$7.1\times 10^{1}$ $5.7\times 10^{0}$ $4.3\times 10^{0}$ $3.6\times 10^{0}$ $2.4\times 10^{0}$ $1.9\times 10^{0}$ $1.8\times 10^{0}$ $1.5\times 10^{0}$ $3.6\times 10^{-1}$ $2.3\times 10^{-1}$ $1.7\times 10^{-1}$ - $G_{f}$ = $9.3\times 10^{1}$	$1.1\times 10^{-5}$ $3.2\times 10^{-4}$ $1.8\times 10^{-3}$ $8.9\times 10^{-3}$ $5.4\times 10^{-2}$ $2.5\times 10^{-1}$ $1.6\times 10^{0}$ $1.6\times 10^{1}$ $9.2\times 10^{1}$ $4.6\times 10^{2}$ $3.0\times 10^{3}$ - $\lambda_{f}$ = $7.2\times 10^{0}$	$7.7\times 10^{-4}$ $1.8\times 10^{-3}$ $7.7\times 10^{-3}$ $3.2\times 10^{-2}$ $1.3\times 10^{-1}$ $4.9\times 10^{-1}$ $3.0\times 10^{0}$ $2.3\times 10^{1}$ $3.3\times 10^{1}$ $1.1\times 10^{2}$ $5.0\times 10^{2}$ $1.0\times 10^{-3}$ $\eta_{0}$ = $6.7\times 10^{2}$
	Human blood with $\phi_{href}=57.60\%$		
1 2 3 4 Plasma Estimated	$4.4\times 10^{-1}$ $5.3\times 10^{-2}$ $6.1\times 10^{-3}$ $1.4\times 10^{-2}$ - $G_{f}$ = $5.2\times 10^{-1}$	$3.1\times 10^{-2}$ $4.5\times 10^{-1}$ $2.3\times 10^{0}$ $2.4\times 10^{1}$ - $\lambda_{f}$ = $7.7\times 10^{-1}$	$1.4\times 10^{-2}$ $2.4\times 10^{-2}$ $1.4\times 10^{-2}$ $3.5\times 10^{-1}$ $4.0\times 10^{-3}$ $\eta_{0}$ = $4.0\times 10^{-1}$

**Table 2:** The zero-shear viscosity, the mean characteristic time and the mean modulus estimated from the fitted master curves of *Phormidium* suspension and human blood with respect to various reference volume fraction.

Sample	$\phi_{\left(h\right)}$ (vol%)	$\eta_{0}$ (Pa·s)	$\lambda_{f}$ (s)	$G_{f}$ (Pa)
*Phormidium* suspension	8.2 10.6 18.0 42.0 60.0	$5.4\times 10^{0}$ $5.7\times 10^{1}$ $6.7\times 10^{2}$ $4.7\times 10^{4}$ $8.0\times 10^{3}$	$1.2\times 10^{-1}$ $1.2\times 10^{0}$ $7.2\times 10^{0}$ $2.1\times 10^{2}$ $1.8\times 10^{1}$	$4.7\times 10^{1}$ $4.9\times 10^{1}$ $9.3\times 10^{1}$ $2.2\times 10^{2}$ $4.6\times 10^{2}$
Human blood	39.80 48.68 57.60 73.83 84.52	$1.9\times 10^{-1}$ $3.1\times 10^{-1}$ $4.0\times 10^{-1}$ $4.1\times 10^{-1}$ $5.4\times 10^{-1}$	$8.4\times 10^{-1}$ $7.1\times 10^{-1}$ $7.7\times 10^{-1}$ $5.2\times 10^{-1}$ $4.0\times 10^{-1}$	$2.2\times 10^{-1}$ $4.4\times 10^{-1}$ $5.2\times 10^{-1}$ $7.9\times 10^{-1}$ $1.4\times 10^{0}$

**Table 3:** The concentration scaling exponents α, −β and α−β of the shifting factors $a_{c}$, $b_{c}^{-1}$ and $a_{c}/b_{c}$, respectively. $\phi_{ref}=1.04\%$ for dsDNA-SWCNT, $\phi_{ref}=6.3\%$ for CNC suspension, $c_{ref}=3wt\%$ for HPC solution, $c_{ref}=56c^{*}$ for PB-PHO, $c_{ref}=48c^{*}$ for 18M PAAm. $M_{w}$ and $M_{n}$ are the number and weight averaged molecular weight of polymer, respectively.

Sample	$L_{w}/L_{n}$ ($M_{w}/M_{n}$)	$\phi /\phi_{(h)\mathrm{ref}}$ ($c/c_{\mathrm{ref}}$)	α	−β	α−β
*Phormidium* suspension	1.14	I: 0.36∼0.46 II: 0.59∼2.89 III: 3.11∼3.44	2.7±0.3 3.8±0.1 12.1±0.6	7.4±0.5 1.03±0.06 9.8±0.3	10.2±0.2 4.9±0.2 21.9±0.5
Human blood		0.69∼1.63	−1.1±0.2	2.4±0.3	1.3±0.2
dsDNA-SWCNT [28] dispersion		II: 0.82∼1.86 III: 2.13∼5.3	3.9±0.4 7.8±0.2	3.6±0.4 6.6±0.4	7.5±0.2 14.4±0.4
CNC [19] suspension	<1.22	II: 0.79∼1.65	9.0±0.5	0.23±0.02	9.2±0.5
HPC [21] solution		0.67∼2	1.5±0.2	0.8±0.2	2.3±0.4
PB-PHO [26]	<1.1	0.19∼7.14	2.45±0.06	2.12±0.02	4.57±0.06
18M PAAm [26]	>34.4	0.21∼5.21	1.40±0.06	1.05±0.03	2.48±0.09

## Data Availability

Not applicable.
